# Serum complement C4b, fibronectin, and prolidase are associated with the pathological changes of pulmonary tuberculosis

**DOI:** 10.1186/1471-2334-14-52

**Published:** 2014-01-31

**Authors:** Chong Wang, Yan-Yuan Li, Xiang Li, Li-Liang Wei, Xiu-Yun Yang, Dan-Dan Xu, Ting-Ting Jiang, Zhong-Jie Li, Zhong-Liang Chen, Xing Zhang, Ji-Yan Liu, Ze-Peng Ping, Ji-Cheng Li

**Affiliations:** 1 Institute of Cell Biology, Zhejiang University, Hangzhou 310058, P.R. China; 2 Department of Pathology, First Affiliated Hospital, Zhejiang University, Hangzhou 310003, P.R. China; 3 Key Laboratory of Gastroenteropathy, Zhejiang Province People’s Hospital, Hangzhou 310012, P.R. China; 4 The Sixth Hospital of Shaoxing, Shaoxing 312000, P.R. China; 5 Department of Respiratory Medicine, Tongde Hospital of Zhejiang Province, Hangzhou 310012, P.R. China

**Keywords:** Serum, Pulmonary tuberculosis, Complement C4b, Fibronectin, Prolidase, Granuloma, Cavity

## Abstract

**Background:**

*Mycobacterium tuberculosis* infection can activate the immune system, leading to characteristic pathological changes such as inflammatory granuloma, caseous necrosis, and cavity formation.

**Methods:**

Clinical data of 187 cases of pulmonary tuberculosis (PTB) were analyzed using statistical methods, while serum levels of complement C4b (C4b), fibronectin (FN), and prolidase (PEPD) were detected using the ELISA method among the control, minimal PTB, moderate PTB, and advanced PTB groups.

**Results:**

We found significantly higher levels of serum C4b and PEPD (*P* = 0.018, *P* = 0.003), and significantly lower levels of serum FN (*P* < 0.001) in PTB patients. Furthermore, the serum levels of 3 proteins were significantly different among 3 PTB groups. FN level was significantly higher in the moderate PTB group, compared with patients in the minimal and advanced PTB groups (*P* < 0.05, *P* < 0.01). PEPD level was significantly higher in the moderate PTB group, compared with the minimal PTB group (*P* < 0.05). Analysis of clinical data showed that serum albumin, C-reactive protein (CRP), prealbumin, and C4 were significantly higher (*P* < 0.05), while serum globulin was significantly lower in patients with PTB (*P* < 0.001). A significant negative correlation was found between C4b and albumin, prealbumin. On the other hand, a significant positive correlation was found between C4b and globulin, CRP, PEPD, as well as between PEPD and CRP (*P* < 0.05).

**Conclusions:**

Our study showed that C4b, FN, and PEPD are associated with tissue damage, granuloma formation, and cavity formation, respectively, in patients with PTB. The present study provides a new experimental basis to understand the pathogenesis and pathological changes of PTB.

## Background

China has the world’s second largest tuberculosis (TB) epidemic with 0.9 to 1.1 million new cases and 45,000 to 49,000 death cases in 2011 [1]. The fifth national TB epidemiological survey data showed that China’s active TB population is up to 4.99 million, including 0.72 million sputum smear positive patients and 1.29 million sputum culture positive patients [2]. We found that the number of active TB patients was even larger due to the increase in the total population compared to 2000, indicating that TB is still a major infectious disease in China.

Pulmonary tuberculosis (PTB) is caused by infection of *Mycobacterium tuberculosis* (MTB). It is one of the most common infectious diseases in the world. Innate immunity provides the first line of defense against MTB infection. In addition, complement proteins also act as a functional bridge between innate and adaptive immunity, thereby participating in many complex immune responses [3]. Several previous studies have shown that defects in innate immunity could lead to PTB progression [4,5] and defensins could cause increased tissue damage [6] while, the Toll-like receptor, CD14 is required for MTB-cell recognition [7,8]. As a part of complement system, the mannose-binding lectin (MBL) pathway can cause cytolysis by identifying mannose residues on the surface of MTB, and complement C4b (C4b) is a product of activated complement C4 (C4) in the early stage of MBL pathway [9]. So, we hypothesized that the C4b levels may be associated with MTB infection and tissue damage. It is well known that PTB undergo many characteristic changes such as granuloma formation, caseous necrosis, and cavity formation, but the molecular mechanisms underlying these changes remain unclear. Currently, many proteins have been demonstrated to participate in the pathogenesis and pathological changes of PTB, including a large amount of extracellular matrix proteins such as matrix metalloproteinase 9 (MMP-9) [10], tissue inhibitor of metalloproteinases-2 (TIMP-2) [11], and osteopontin [12]. MMP-9 has been shown to be involved in the recruitment of macrophages and tissue remodeling at the early stage of granuloma formation in PTB [10]. Fibronectin (FN) is a type of extracellular matrix proteins, which binds to β1 integrin on the cell surface, leading to cellular adhesion to the extracellular matrix. Considering this, there is a big chance that FN could be involved in granuloma formation. TB cavity is formed by liquid discharge through the bronchial tree after the hard caseum softens [13]. Kumar et al. [14] attributed granuloma formation, caseous necrosis, and liquefaction to host proteases disorder. Protease (PEPD) is a type of proteases that hydrolyzes peptides with proline or hydroxyproline at the carboxy terminus. All together, we hypothesized that serum C4b (Swiss-Prot: P20851), FN (Swiss-Prot: P02751), and PEPD (Swiss-Prot: P12955) levels may be associated with MTB infection, tissue damage, granuloma formation, cavity formation and other pathological changes in PTB patients.

In this study, we explored the serum C4b, FN, and PEPD levels in patients with PTB and healthy controls. We divided PTB patients according to the standard of the modified classification of the National Tuberculosis Association (NTA) of the USA and revealed the relationship between the three proteins and pathological changes in order to clarify the role of these proteins in the pathogenesis of clinical TB.

## Methods

### Patients and control subjects

A total of 187 subjects with pulmonary tuberculosis were recruited from the Sixth Hospital of Shaoxing. A total of 115 subjects, aged 18–70 years (mean age 41.6 ± 17.2 years) were tested by ELISA. The control group comprised 39 healthy subjects, aged 23–58 years (mean age 39.9 ± 9.9 years), and unrelated blood donors with no history of TB or other immune diseases. Females constituted 31.3% of the PTB patients, and 38.5% of healthy controls (Table 1). This study was approved by the Ethics Committee of the Faculty of Medicine (Zhejiang University, China), and informed consent was obtained from all subjects before blood sampling. Blood was drawn into regular bottles in the morning from each patient before the anti-TB therapy. Similarly, fasting blood samples were drawn from healthy controls. The samples were stored at - 70°C for further analysis.

**Table 1:** Characteristics of pulmonary tuberculosis patients and healthy controls

	**PTB patients (N = 115)**	**Controls (N = 39)**	***P***
Age, years range (mean ± SD)	18−70 (41.6 ± 17.2)	23−58 (39.9 ± 9.9)	0.582^a^
Gender: female, no. (%)	36 (31.3)	15 (38.5)	0.711^b^
Body mass index (mean ± SD)	21.3 ± 3.2	23.2 ± 4.3	0.213^a^
Tuberculin skin test (>10 mm), no. (%)	66 (57.4)	ND	/
Positive sputum smears, no. (%)	63 (54.8)	ND	/
Presence of TB history of relatives, no. (%)	11 (9.6)	3 (7.7)	0.747^b^
BCG vaccination, no. (%)	48 (41.7)	21 (53.8)	0.426^b^

A *P*-value of less than 0.05 indicates statistical significance. PTB: pulmonary tuberculosis; N: number of subjects; ND: not determined. ^a^*P* value between PTB patients and controls, for *t* test. ^b^*P* value between PTB patients and controls, for χ^2^ test.

Patients were diagnosed according to the diagnostic criteria for PTB of Ministry of Health of the People’s Republic of China [15]. All patients meet one of the following PTB diagnostic criteria: (1) positive sputum examination (smear or culture); (2) negative sputum examination, chest X-ray, and CT revealing evidence of typical active TB; (3) pathological diagnosis of TB in lung specimens; (4) suspected of having PTB after clinical follow-up and X-ray observations, and excluding other lung diseases; (5) clinically ruling out other causes of pleural effusion, and diagnosis of tuberculous pleurisy. All patients were classified as having minimal, moderate or advanced PTB using a modified classification of the NTA [16,17]. The study group comprised 115 PTB patients classified as minimal (N = 39), moderate (N = 41), or advanced (N = 35) PTB. There was no significant difference in the age and gender distribution among the three groups.

### ELISA methods

Human C4b ELISA Kit (Cusabio Biotech Co., LTD, China), with a detection limit of 15.6 ng/mL, was used to detect C4b in serum. Human FN ELISA Kit (Abnova Co., Taipei, Taiwan), with a minimum detection limit of 0.31 ng/mL, was used to detect FN in serum. Human PEPD ELISA kit (Cusabio Biotech. Co., LTD, China), with a minimum detection limit of 93.75 mU/mL, was used to detect PEPD in serum. The protein concentration of 39 healthy controls and 115 PTB patients were measured according to the manufacturer’s instructions. Briefly, serum samples were diluted with dilution factors of 1:2,000, 1:1,000,000 and 1:10 for C4b, FN and PEPD, respectively. Diluted samples were incubated in microtiter wells coated with antibodies of proteins. After incubation and washing, the biotinylated tracer antibody conjugated with streptavidin-peroxidase was added to the wells. Substrate tetramethylbenzidine (TMB) was added to the wells after a second incubation and washing, and then the oxalic acid was added to stop the enzyme reaction. Absorbance was read on xMark microplate spectrophotometer (Bio-Rad, Inc., USA) at a wavelength of 450 nm. The concentration of the protein was estimated using a four-parameter logistic curve (Microplate Manager 6 software, Bio-Rad, Inc., USA) based on the measured standard values.

### Statistical analysis

Parametric data were presented as mean ± SD while nonparametric data were presented as median ± IQR, and *P* < 0.05 was considered as statistically significant by the SPSS software, version 16.0 (SPSS, Chicago, IL). The One-sample *t*-test was used to investigate the difference between PTB patients and normal reference range after taking the logarithm. Two groups’ data were tested using the chi-square test for composition ratio and *t*-test for means. Nonparametric analysis was carried out using the Mann–Whitney U-test. Spearman correlation method was performed to determine association between two different parameters. The study sample provided 88.93% power to identify significant differences between whole PTB patients and healthy controls at a statistical support level of α = 0.05 with an d of 0.6 applying a two tails model, and provided 75.47% power when identifying significant differences between minimal, moderate, advanced PTB groups and healthy controls.

## Results

**Figure 1:**
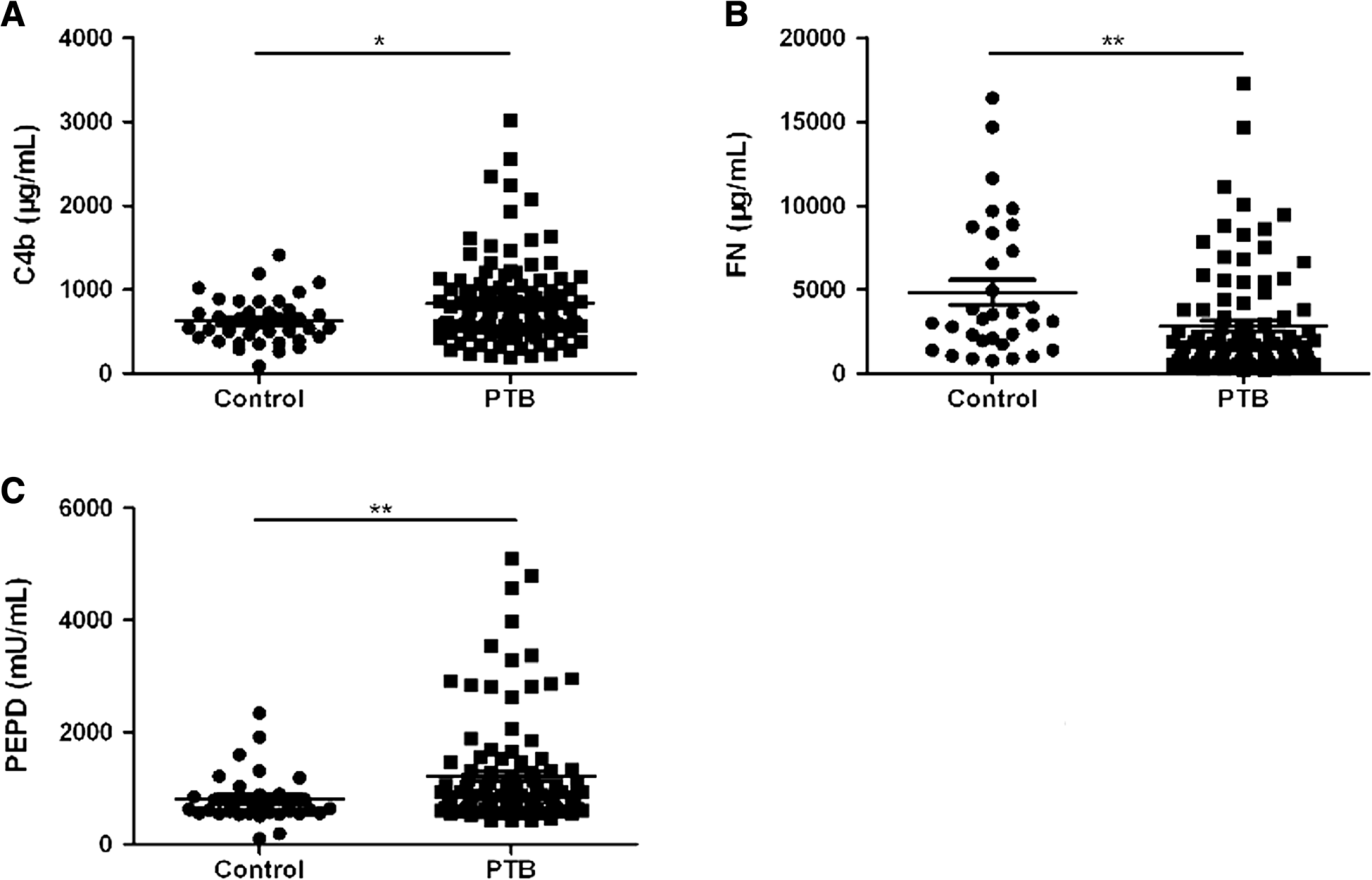
**Serum C4b, FN, and PEPD levels between controls and pulmonary tuberculosis patients.** A *P*-value of less than 0.05 indicates statistical significance using the Mann–Whitney U-test. PTB: pulmonary tuberculosis; ^*^P < 0.05; ^**^P < 0.01. **A**: the levels of C4b; **B**: the levels of FN; **C**: the levels of PEPD.

Clinical data analysis of 187 PTB cases showed various significant differences between PTB patients and healthy controls of different parameters (Table 2). Comparison of 115 PTB patients and 39 healthy controls by using the ELISA method showed significant higher levels of serum C4b and PEPD (*P* = 0.018, *P* = 0.003), and significant lower level of FN (*P* < 0.001) in PTB patients (Figure 1).

**Table 2:** Clinical data of pulmonary tuberculosis patients (N = 187) and normal reference ranges

	**PTB patients (mean ± SD)**	**Normal reference ranges**	**Median**	***P***^**a**^
Total protein (g/L)	68.32 ± 6.66	64.00 − 83.00	72.88	< 0.001^**^
Albumin (g/L)	37.94 ± 5.13	36.00 − 52.00	43.27	< 0.001^**^
Globulin (g/L)	30.27 ± 5.60	22.00 − 34.00	27.35	< 0.001^**^
A/G	1.29 ± 0.32	1.20 − 2.50	1.73	< 0.001^**^
CRP (mg/L)	29.69 ± 38.72	0.00 − 0.82	0.41	< 0.001^**^
Prealbumin (g/L)	0.18 ± 0.08	0.15 − 0.36	0.23	< 0.001^**^
Fibrinogen (g/L)	5.69 ± 2.23	1.90 − 5.60	3.26	< 0.001^**^
Triglyceride (mmol/L)	1.06 ± 0.67	0.56 − 1.69	1.05	0.001^**^
LDH (U/L)	167.55 ± 54.74	100.00 − 240.00	154.92	0.064
HDL-C (mmol/L)	1.03 ± 0.34	1.03 − 1.55	1.26	< 0.001^**^
LDL-C (mmol/L)	2.31 ± 0.81	0.00 − 3.10	1.55	< 0.001^**^
Lipoprotein (mg/L)	270.60 ± 275.90	0.00 − 300.00	150.00	0.004^**^
APOA1 (g/L)	1.05 ± 0.23	1.20 − 1.60	1.39	< 0.001^**^
APOB (g/L)	0.76 ± 0.20	0.80 − 1.05	0.92	< 0.001^**^
IgG (g/L)	14.05 ± 3.60	11.50 − 14.22	12.79	0.007^**^
IgA (g/L)	2.84 ± 1.37	1.70 − 3.25	2.35	0.044^*^
IgM (g/L)	1.21 ± 0.57	0.73 − 1.17	0.92	< 0.001^**^
Complement 4 (mg/L)	377.78 ± 80.38	100.00 − 400.00	200.00	< 0.001^**^
Complement 3 (g/L)	1.04 ± 0.24	0.83 − 1.77	1.21	< 0.001^**^

A *P*-value of less than 0.05 indicates statistical significance. PTB: pulmonary tuberculosis; N: number of subjects; A/G: Albumin/ Globulin; CRP: C-reactive protein; LDH: Lactate dehydrogenase; HDL-C: High-density lipoprotein cholesterol; LDL-C: Low-density lipoprotein cholesterol; APOA1: apolipoprotein A1; APOB: apolipoprotein B; ^*^P < 0.05; ^**^P < 0.01. ^a^*P* value between TB patients and normal reference range, for one-sample *t*-test after taking the logarithm then compare to the median.

**Figure 2:**
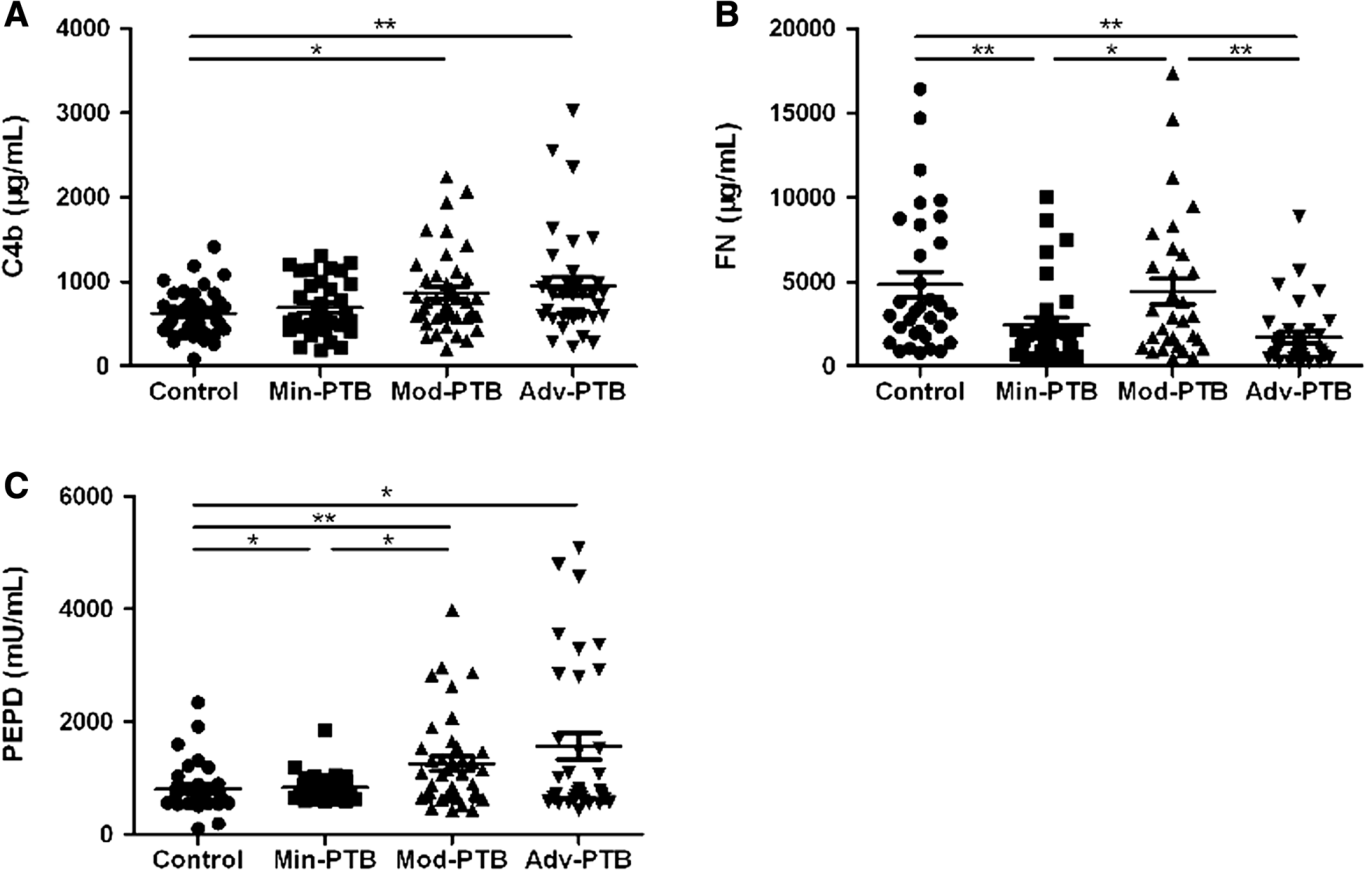
**Serum C4b, FN, PEPD levels between controls and pulmonary tuberculosis patients classified by NTA classification.** A *P*-value of less than 0.05 indicates statistical significance using the Mann–Whitney U-test. Min-PTB: minimal PTB; Mod-PTB: moderate PTB; Adv-PTB: advanced PTB; ^*^P < 0.05; ^**^P < 0.01. **A**: the levels of C4b; **B**: the levels of FN; **C**: the levels of PEPD.

Then, we compared the control group with the minimal, moderate, and advanced PTB groups. Our results indicated that the levels of serum C4b were different among groups (*P* = 0.024). There were significant differences in serum C4b levels of the moderate and advanced PTB groups, compared to the control group (*P* < 0.05, *P* < 0.01) (Figure 2A). We also found different levels of FN (*P* < 0.001) among groups. FN level was found to be significantly different in the moderate PTB group, compared with patients in the minimal and advanced PTB groups (*P* < 0.05, *P* < 0.01), not to mention the control and minimal (*P* < 0.01), and the control and advanced PTB groups (*P* < 0.01) (Figure 2B). PEPD also showed different expression levels among the groups (*P* = 0.011). There were significant differences in serum PEPD levels between all three PTB groups and the control group (*P* < 0.05, *P* < 0.01, *P* < 0.05). In addition, a significant higher PEPD level was found in the moderate PTB group than the minimal PTB group (*P* < 0.05) (Figure 2C).

**Figure 3:**
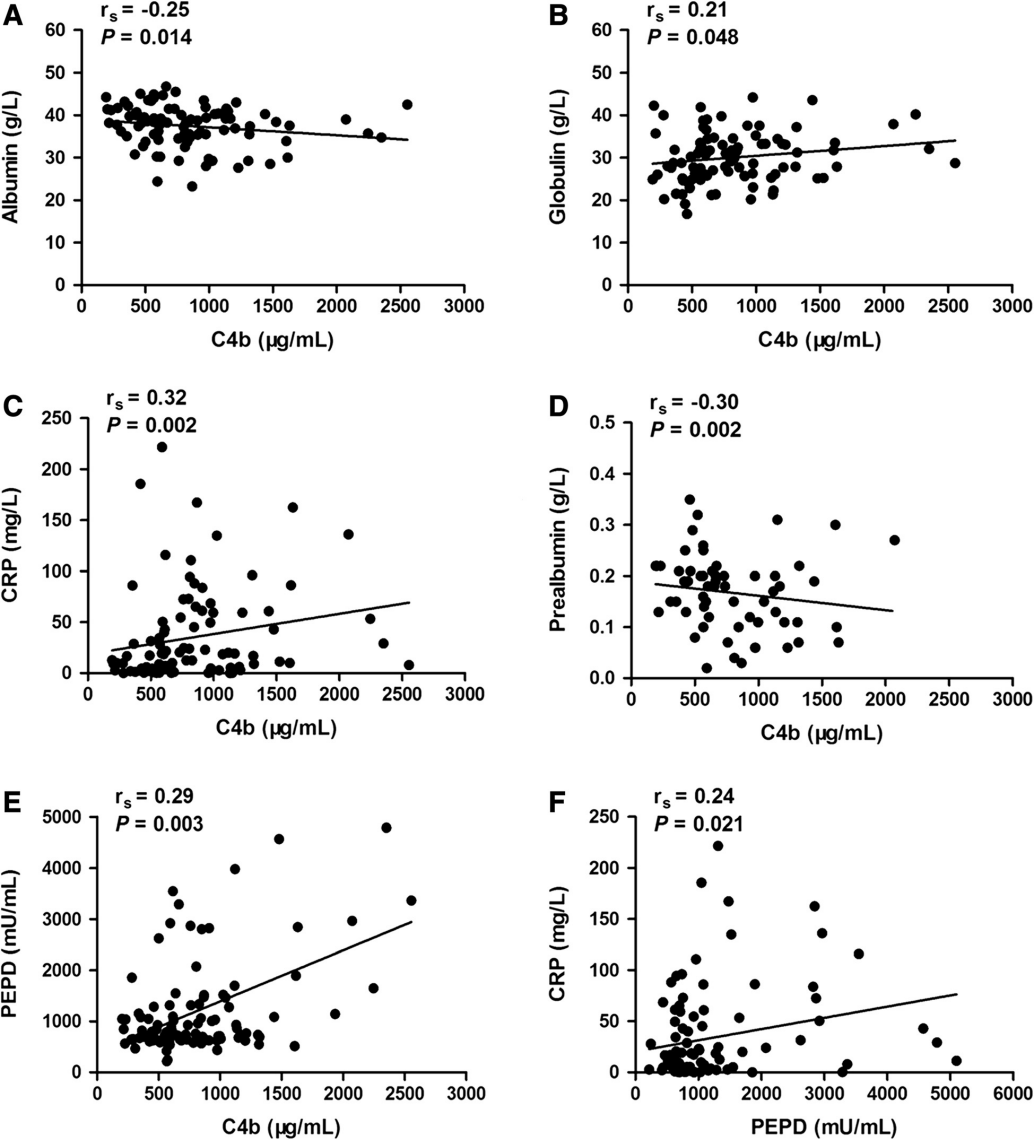
**Correlations between serum C4b, PEPD and some biochemical parameters.** A *P*-value of less than 0.05 indicates statistical significance using the Spearman correlation method. The range of r_s_ from −0.3 to −0.1 or from 0.1 to 0.3 means weak correlation, while the range from −0.5 to −0.3 or from 0.3 to 0.5 means moderate correlation. **A**: correlation of C4b and albumin; **B**: correlation of C4b and globulin; **C**: correlation of C4b and C-reactive protein; **D**: correlation of C4b and prealbumin; **E**: correlation of C4b and PEPD; **F**: correlation of PEPD and C-reactive protein.

By using the Spearman correlation analysis, we found a significant negative correlation between C4b and albumin, prealbumin. A significant positive correlation was observed between C4b and globulin, CRP, PEPD, as well as PEPD and CRP (*P* < 0.05) (Figure 3). After that, we separated all 115 subjects based on age, gender, current smoker, single/double lung lesion, cavity/non-cavity, sputum smear results (−/+/++/+++/++++). Significant difference of C4b level was found between smokers and non-smokers (*P* = 0.012), as well as PEPD level between males and females (*P* = 0.011) (Table 3). In addition, FN was related to sputum smear positive results (r_s_ = 0.22, *P* = 0.028) (Table 4).

**Table 3:** Average serum levels of proteins according to the clinical characteristics of the pulmonary tuberculosis patients

**Clinical characteristics (Cases)**	**C4b (μg/mL)**	***P***^**a**^	**FN (μg/mL)**	***P***^**a**^	**PEPD (mU/mL)**	***P***^**a**^
**Age**		0.651		0.139		0.499
18−34 (51)	663.83 ± 482.42		1,931.50 ± 3,389.50		784.47 ± 492.87	
35−49 (19)	610.22 ± 539.22		837.00 ± 1,338.00		764.53 ± 724.93	
≥50 (45)	772.48 ± 508.49		1,567.00 ± 2,292.00		931.92 ± 647.87	
**Gender**		0.698		0.545		0.011^*^
Male (79)	694.83 ± 525.59		1,637.50 ± 3,085.25		878.56 ± 628.99	
Female (36)	747.14 ± 417.058		1,274.00 ± 2,198.00		672.24 ± 341.35	
**Current smoker**		0.012^*^		0.838		0.205
Yes (50)	614.25 ± 464.86		1,582.50 ± 3,446.25		925.33 ± 635.58	
No (65)	818.59 ± 471.98		1,511.00 ± 2,285.50		778.75 ± 557.74	
**Lung lesion**		0.320		0.692		0.357
Single (53)	645.45 ± 444.61		1,550.00 ± 1,705.00		778.75 ± 380.34	
Double (62)	808.42 ± 454.55		1,582.50 ± 3,508.75		845.27 ± 856.62	
**Chest X-ray**		0.555		0.739		0.878
Non-cavity (70)	663.83 ± 664.71		1,567.00 ± 1,675.00		833.59 ± 405.02	
Cavity (45)	729.10 ± 389.30		1,637.50 ± 3,566.75		755.98 ± 871.74	
**Sputum smear**		0.258		0.121		0.441
Negative (52)	645.45 ± 526.00		1,509.00 ± 1,925.75		771.10 ± 326.47	
1+ (13)	587.64 ± 326.10		1,189.00 ± 1,348.00		1,079.22 ± 810.94	
2+ (18)	976.66 ± 500.99		1,725.00 ± 2,333.00		819.06 ± 674.19	
3+/4+ (32)	791.67 ± 409.65		2,504.00 ± 3,974.50		925.33 ± 833.83	
**NTA classification**		0.024^*^		< 0.001^**^		0.011^*^
Minimal PTB (39)	567.63 ± 518.38		1,528.00 ± 1,605.00		795.95 ± 258.84	
Moderate PTB (41)	729.10 ± 472.71		2,894.00 ± 4,915.00		968.67 ± 759.27	
Advanced PTB (35)	846.87 ± 402.95		856.00 ± 1,609.00		761.75 ± 1,895.57	

All the data were presented as median ± IQR. A *P*-value of less than 0.05 indicates statistical significance. ^a^*P* value among groups, for Mann–Whitney U-test. ^*^P < 0.05; ^**^P < 0.01.

**Table 4:** Correlations for clinical characteristics and serum protein levels of the pulmonary tuberculosis patients (N = 115)

		**Sputum smear**	**NTA classification**	**C4b**	**FN**	**PEPD**
Sputum smear	r_s_	1	0.19	0.11	0.22	0.10
	*P*^a^	.	0.043^*^	0.244	0.028^*^	0.287
NTA classification	r_s_		1	0.19	−0.18	0.08
	*P*^a^		.	0.045^*^	0.086	0.420
C4b	r_s_			1	0.10	0.29
	*P*^a^			.	0.354	0.003^**^
FN	r_s_				1	−0.05
	*P*^a^				.	0.659
PEPD	r_s_					1
	*P*^a^					.

The r_s_ in [−0.3, -0.1] or [0.1, 0.3] means weak correlation, in [−0.5, -0.3] or [0.3, 0.5] means moderate correlation, and in [−0.7, -0.5] or [0.5, 0.7] means significant correlation. A *P*-value of less than 0.05 indicates statistical significance. N: number of subjects; ^a^*P* value for Spearman Correlation. ^*^P < 0.05; ^**^P < 0.01.

## Discussion

Innate immunity is the body’s first line of defense against MTB infection. As a part of the complement system, the MBL pathway can cause cytolysis by identifying mannose residues on the surface of MTB. C4b is a product of activated C4 in the early stage of MBL pathway [9]. Clinical data analysis showed liver dysfunction (decreased albumin), immune system activation (increased globulin), and complement system activation (increased C4, C3) in PTB patients (Table 2). Similar findings were observed by ELISA method (Figure 1A). Correlation analysis demonstrated a weak but significant correlation between C4b and albumin, globulin (*P* < 0.05) (Figure 3A, 3B), indicating that the reduction of liver synthetic function, and the activation of immune and complement systems occurred in the early stage of PTB. Kingery et al. [18] revealed that increased plasma C4b may enhance the tissue damage by inflammatory response, indicating C4b as a prognostic biomarker of type I diabetes. In our study, a significant difference in C4b expression was observed in the moderate and advanced PTB patients, compared to the control group (*P* < 0.05), suggesting that a larger amount of bacterial infection may cause complement system activation, tissue damage, and cavity formation, which is also proven by Yoon et al. [19].

PTB is characterized by necrotizing granulomatous inflammation of the lung tissue. FN is an extracellular glycoprotein that binds to β1 integrin on the cell surface, leading to cellular adhesion to the extracellular matrix. There are two types of FN: plasma fibronectin (pFN), a soluble dimer present in the body fluid, and cellular fibronectin (cFN), an insoluble oligomer present in the extracellular matrix [20]. Fibroblasts could secrete proteases, such as metalloproteinase to digest pFN at first, and then secrete cFN to form extracellular matrix during the tissue injury. This may be the reason for the decreased serum FN levels (*P* < 0.001) in PTB patients in our study. Moreover, Kim et al. [21] revealed increased serum pFN in retinoic acid-deficient mice, which could be reversed by adding exogenous retinoic acid. Retinol-binding protein 4 (RBP4) is responsible for binding and transporting blood retinol into the cells to become retinoic acid, and RBP4 reduction was observed previously in PTB patients [22]. Therefore, we hypothesized that reduced RBP4 protein may be related to the FN reduction in PTB patients. RBP4 transport retinol into the cell, resulting in increased intracellular retinoic acid and decreased serum FN.

Interestingly, there was a higher level of FN in the moderate PTB group, compared to both minimal and advanced PTB groups (*P* < 0.05, *P* < 0.01). FN is thought to be the earliest biomarker of *Schistosoma haematobium* and *Schistosoma mansoni* infested granuloma [23]. Rojas et al. [24] demonstrated that phosphatidylinositol mannoside of MTB can bind to α_5_β_1_ integrin on CD4+ T cells, resulting in T cell adhesion to FN in granuloma. So, we suggested that a higher FN level in moderate PTB patients is related to the granuloma formation. In addition, correlation between FN and sputum smear positive results (r_s_ = 0.22, *P* = 0.028) supported our suggestion. As the disease progresses, advanced PTB patients had a reduced level of serum FN, which may be caused by the increased expression of proteases, resulting in increased FN digestion.

Host proteases disorders can cause granuloma formation, caseous necrosis, and liquefaction, leading to collagen degradation and tissue damage. PEPD is a type of proteases that hydrolyzes peptides with proline or hydroxyproline at the carboxy terminus. Myara et al. [25] demonstrated a higher plasma PEPD activity in the early stage of chronic liver disease, indicating its role in the extracellular matrix formation. Gumus et al. [26] found a higher serum PEPD activity in patients with PTB, especially in patients with the lung cavity. It was suggested that increased PEPD level might be related to tissue damage, enhanced fibroblast activity, and increased levels of immunoglobulin and complement C1q (C1q). However, our previous unpublished results, using iTRAQ 2D LC-MS/MS technique, showed no significant difference in serum C1q level between PTB patients and the control group. Clinical data analysis showed a higher levels of IgG, IgA, and IgM in patients with PTB (*P* < 0.05) (Table 2), supporting the speculation that high immunoglobulin levels can lead to increased PEPD level. Therefore, we hypothesized that increased serum PEPD level might be related to tissue damage, enhanced fibroblastic activity, and increased immunoglobulin. Significantly higher levels of PEPD in moderate PTB patients, compared to minimal PTB patients (*P* < 0.05) indicated that PEPD may be involved in the granuloma substrate hydrolysis process leading to the cavity formation.

Furthermore, there was a significant difference in C4b level between smokers and non-smokers (*P* = 0.012), and a significant difference was observed in PEPD level between males and females (*P* = 0.011). We suggested that the lower level of C4b in current smokers may be caused by a reactive inhibition induced by the activation of complement system than non-smokers before MTB infection. The reason for higher level of PEPD in males may be due to the higher ratio of hepatitis B infection among males leading to liver fibrosis [27], as PEPD is an early indicator of liver fibrosis [25].

## Conclusion

In conclusion, we revealed increased serum C4b and PEPD levels, and decreased FN level in PTB patients. C4b may be associated with tissue damage, while FN and PEPD are associated with granuloma and cavity formation, respectively. The study provides a new experimental basis to understand the pathogenesis and pathological changes of PTB.

## Competing interests

The authors declare they have no competing financial interests.

## Authors’ contributions

LJC conceived the study and designed the experiments. WC, LYY, LX, WLL, YXY, LZJ, CZL, ZX, LJY and PZP collected the serum samples and clinical data. WC, XDD and JTT analyzed the data with suggestions by LJC. WC and LJC wrote the manuscript. All authors read and approved the final manuscript.

## Pre-publication history

The pre-publication history for this paper can be accessed here:

http://www.biomedcentral.com/1471-2334/14/52/prepub
